# Optimization of Physiochemical Parameters during Bioremediation of Synthetic Dye by* Marasmius cladophyllus* UMAS MS8 Using Statistical Approach

**DOI:** 10.1155/2016/8296239

**Published:** 2016-10-10

**Authors:** Fatin Nur Sufinas Shuib, Ahmad Husaini, Azham Zulkharnain, Hairul Azman Roslan, Tay Meng Guan

**Affiliations:** ^1^Department of Molecular Biology, Faculty of Resource Science and Technology, Universiti Malaysia Sarawak, 94300 Kota Samarahan, Sarawak, Malaysia; ^2^Department of Chemistry, Faculty of Resource Science and Technology, Universiti Malaysia Sarawak, 94300 Kota Samarahan, Sarawak, Malaysia

## Abstract

In many industrial areas such as in food, pharmaceutical, cosmetic, printing, and textile, the use of synthetic dyes has been integral with products such as azo dye, anthrax, and dyestuffs. As such, these industries produce a lot of waste by-products that could contaminate the environment. Bioremediation, therefore, has become an important emerging technology due to its cost-sustainable, effective, natural approach to cleaning up contaminated groundwater and soil via the use of microorganisms. The use of microorganisms in bioremediation requires the optimisation of parameters used in cultivating the organism. Thus the aim of the work was to assess the degradation of Remazol Brilliant Blue R (RBBR) dye on soil using Plackett-Burman design by the basidiomycete,* M. cladophyllus* UMAS MS8. Biodegradation analyses were carried out on a soil spiked with RBBR and supplemented with rice husk as the fungus growth enhancer. A two-level Plackett-Burman design was used to screen the medium components for the effects on the decolourization of RBBR. For the analysis, eleven variables were selected and from these four parameters, dye concentration, yeast extract concentration, inoculum size, and incubation time, were found to be most effective to degrade RBBR with up to 91% RBBR removal in soil after 15 days.

## 1. Introduction

Synthetic dyes are chemicals that are important alternatives in many industries. Since 1856, synthetic dyes have been used as they are economical to produce and create brighter, more colour-fast, and easy applications. However, most industrial dye processes involve the dye solution to be released to the surrounding water and soils. It has been reported that about 10–15% of all dyestuff is directly lost to wastewater [1] because of inadequate treatment of wastewater and poor waste management. Thus, this will lead to dye contamination in soil and water bodies [2]. Azo, anthraquinone, and phthalocyanine are the most common groups of synthetic dyes used [3]. Remazol Brilliant Blue R (RBBR) is a compound that is normally used to analyse azo dye degradation in laboratory condition [4]. RBBR, or Reactive 19, is an anthraquinone-based dye [5] which is an important dye in textile industries. It also represents a class of toxic and recalcitrant organopollutants that has identical structure to some polycyclic aromatic hydrocarbons (PAH) [6]. RBBR, an anthraquinone-based dye, is not easily degraded even by bacteria due to the presence of the aromatic structure [7]. The chemical structure and specification of the RBBR dye are shown in Table 1 [5].

Among industrial effluents, wastewater from textile and dyestuff industries is one of the most difficult to be treated. This is because industrial textile dyes have been purposely designed to be highly resistant to washing, chemical agents, solvents, and environmental factors such as action by sunlight. The synthetic and complex aromatic molecular structures of synthetic dyes also make them more stable and difficult to be degraded by microbial attack [8].

Conventional wastewater treatment plants using activated sludge treatment are unable to sufficiently treat dye containing wastewater with up to 90% of reactive textile dyes still remaining after the treatment [9]. In another study done by Shaul et al. (1991) [10], 11 out of 18 azo dyes studied were found to pass through activated sludge process substantially untreated while another 4 azo dyes were just adsorbed onto the waste activated sludge. Only 3 dyes (Acid Orange 7, Acid Orange 8, and Acid Red 88) were biodegraded by the activated sludge process.

These environmental pollutants are the contaminants that enter the environment and cause adverse changes. The pollutants can be cleaned up but at a high cost to the people. Furthermore, there are several limitations in the use of physicochemical methods such as adsorption, coagulation, precipitation, filtration, and oxidation [11]. This is because those methods are not environmental friendly and cost competitive compared to bioremediation [12]. Bioremediation on the other hand, by using fungi or bacteria, is an alternative way of cleaning up pollutants [11].

White rot fungi are important and have been used to discover organisms that can degrade synthetic compounds. For example, lignin biodegradation of white rot fungi involves the degradation of aromatic xenobiotics, heterocyclic aromatic hydrocarbons, synthetic high polymers, chlorinated aromatic compounds, and various dyes which are all environmentally persistent pollutants [13]. In the paper and pulp industry, degradation of lignin is needed to eliminate lignin from the wastewater [14]. For the degradation of lignin, white rot fungi produce extracellular enzymes, namely, lignin peroxidase (LiP), manganese peroxidase (MnP), and laccase (Lac), which are involved to effectively depolymerize lignin producing carbon dioxide and water [14, 15]. Apart from that, white rot fungi have a high lignin degradation ability due to their strong oxidative activity and low substrate specificity of their ligninolytic enzymes [14]. Previous studies showed RBBR biodegradation by various fungi such as* Phanerochaete chrysosporium* and* Irpex lacteus*, [4, 16].* Irpex lacteus* is one of white rot fungi that have been reported to be one of the first bioremediation agents of soil [16].

Here we report on the bioremediation of RBBR by* M. cladophyllus* UMAS MS8, in soil and under aerobic conditions. Eleven parameters in the bioremediation process were tested with the aim to determine the optimal parameters. Statistical approaches utilizing Plackett-Burman design were applied to optimize the concentration of dye, concentration of yeast, and incubation time as variables that affected the bioremediation rate.

## 2. Materials and Methods

### 2.1. Microorganism Preparation and Culture Maintenance

*Marasmius cladophyllus *UMAS MS8 was used in this study and obtained from Molecular Biology Laboratory, Universiti Malaysia Sarawak (UNIMAS). The fungus was grown on malt extract agar (MEA) for a period of 7 to 10 days at 29°C. Malt extract agar (MEA) was prepared by dissolving 33.6 gL^−1^ of malt extract in distilled water, with pH 5.6, and autoclaved for 1 h at 121°C. Stock fungus culture was maintained in 20% (v/v) glycerol stored at −20°C, in sterile distilled water and on plates of malt extract agar (MEA) medium added with 0.05% (w/v) chloramphenicol.

### 2.2. Soil and Substrate Preparation

Top soil sample was used and oven-dried for 4 days at 70°C before being used in the experiment. The soil was sieved through 2.0 mm mesh sieve and the physicochemical properties were partially characterized. Results showed that the soil used was clay textured with pH of 4.73 (1 : 5 H_2_O) and contained organic carbon of 70.133 g kg^−1^ (w/w) glucose equivalent, a total nitrogen of 80.06 mg L^−1^, and bioavailable P of 1.96 mg kg^−1^. These indicated a ratio of C : N : P of 35.8 : 40.8 : 1 which was lower than the commonly used C : N : P ratio of 100 : 10 : 1. The sterile soil was prepared by autoclaving three times continuously at 24-hour intervals for 55 min at 121°C.

Rice husk that was used as growth enhancer was air-dried and large particles were removed. Sterile rice husk was prepared by autoclaving for 1 h at 121°C and the moisture content was determined by drying 3 g of rice husk in an oven for three days at 70°C. After three days, rice husk was weighted again until a constant mass was obtained. The total water loss of rice husk also was determined using the same equation as for the soil

(1) $$\begin{array}{c} \%\text{ of Moisture Content}=\frac{\left(\text{Weight of the air-dried RH}\left(g\right)\right)-\left(\text{weight of oven-dried RH}\left(g\right)\right)}{\text{Weight of the air-dried RH}\left(g\right)}\times 100. \end{array}$$

### 2.3. Screening of RBBR Dye Degradation on Agar Medium and Bioremediation Experiments on Soil

*Marasmius cladophyllus* UMAS M8 was screened for RBBR decolourization capability on malt extract agar (MEA) supplemented with 0.02%, 0.06%, and 0.1% (w/v) RBBR dye. The agar plates were inoculated with 1.0 cm^2^ (in diameter) of mycelium plug from a 7-day-old fungal mycelium. The fungal isolate together with the control plate was prepared in triplicate and incubated for 3 to 15 days in the dark at room temperature [17]. Bioremediation experiments were performed in 50 mL of Erlenmeyer flasks with 6 g of dry soil that was homogenously spiked with 0.1% (w/v) RBBR solution. A 30% (w/w) rice husk was used and added onto the top of the soil. The spiked soil was individually treated with 1.0 cm^2^ of fungal mycelial plug with 70% (w/v) water moisture content. Noninoculated flasks with the respective dye concentrations were used as controls. Each culture condition was prepared in triplicate and incubated in the dark at room temperature under static condition. Other parameters were standardized using the experimental design layout.

### 2.4. Screening of Important Nutrient Components Using Plackett-Burman Design

Plackett-Burman design was used for screening the important medium components with respect to their main effects but not their interaction effects on RBBR decolourization. Plackett-Burman design provides an efficient and rapid method to screen and select ingredients with maximum number of variables [17, 18]. A 12-run Plackett-Burman design was applied to this study to evaluate eleven variables at two levels: maximum (+1) and minimum (−1). The design and levels of each variable are shown in Table 2 and all trials were performed in triplicate. The medium was formulated as per the design and the response was calculated as the rate of dye decolourization and expressed in percentage.  Table 3 represented the list of ingredients and concentrations chosen of 12 screening experiments.

The main effect of each variable was determined with the following equation:

(2) $$\begin{array}{c} E_{xi}=\frac{\left(\sum M_{i+}-\sum M_{i-}\right)}{N}, \end{array}$$

where *E*_*xi*_ is variable main effect, *M*_*i*+_ and *M*_*i*−_ are the RBBR degradation percentages in trials where the independent variable (*xi*) was present in high and low concentrations, and *N* is the number of trials divided by 2.

Experimental error was estimated by calculating the variance between the two dummy variables using following equation:

(3) $$\begin{array}{c} V_{eff}=\frac{\sum {\left(E_{d}\right)}^{2}}{n}, \end{array}$$

where *V*_eff_ is the variance of the effect, *E*_*d*_ is the effect for the dummy variable, and *n* is the number of dummy variables used in the experiment.

The standard error (SE) of the effect was the square root of *V*_eff_ and the significance (*p* value) of the effect of each variable on phenol degradation was measured by Student's *t*-test as follows:

(4) Xtt=EXtSE,

where *E*(*X*_*t*_) is the effect of variable *X*_*t*_.

### 2.5. Residual RBBR Extraction and Quantification

Residual RBBR extraction was carried out according to the procedure as described by Novotný et al. [16] with some modification, by using a multisolvent system (chloroform, methanol, distilled water: 1 : 1 : 1, v/v). Each of the solvents was added separately, followed by mixing and vigorous shaking of the soil samples. Then the flasks contents were sonicated for 15 min and filtered through filter paper. Chloroform was separated from the filtrate with the help of micropipette. The filtrate was centrifuged for 10 min at 10,000 rpm, then placed in an open glass petri dish, and allowed to evaporate at 100°C for 6–8 h. The residue was redissolved in 7 mL of ddh_2_O and centrifuged for 10 min at 10,000 rpm. The control, soil without the RBBR, underwent similar procedure. Residual RBBR dye was quantified by measuring the absorbance at 595 nm [16]. Change in colour was observed which provided the rate of biodegradation of dye [19]. Percentage of degraded RBBR dye was calculated using (5) as follows:

(5) $$\begin{array}{c} \text{Percentage of decolourisation}\left(\;\%\;\right)=\frac{Ac-As}{Ac}\times 100, \end{array}$$

where Ac is the absorbance at the maximum absorption wavelength of dye in the control flask at time, *t*, and As is the absorbance at the maximum absorption wavelength of dye in the sample flask at time, *t* [17, 20].

## 3. Results and Discussion

### 3.1. Screening of Degradation Assay in Agar

**Figure 1:**
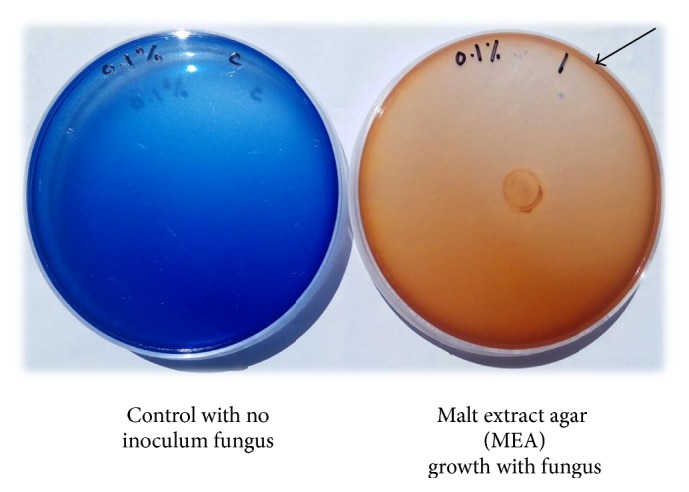
Complete dye decolourization by* M. cladophyllus* UMAS MS8 resulting in the formation of halo (arrow) on agar plate medium containing RBBR.

*Marasmius cladophyllus* UMAS MS8 was used to decolourize RBBR and evaluate the dye decolourization characteristics. This work was carried out on solid agar medium and observation showed that the fungus grew on MEA supplemented with RBBR dye. MEA supplemented with 0.1% (w/v) of RBBR showed complete decolourization with formation of halo on agar plate after 9 days of incubation with 1.0 cm^2^ of mycelial plug (Figure 1). Other results were also recorded and shown in Table 4. The fungal isolate together with the control plate was prepared in triplicate and incubated for a period of 3 to 15 days at 28°C.

### 3.2. Screening Using Plackett-Burman Design

The effects of eleven variables, lactose, glucose, dye concentration, (NH_4_)_2_SO_4_, NH_4_NO_3_, tryptone, yeast, NH_4_Cl, inoculum size, moisture content, and incubation time, supplemented with rice husk on RBBR dye degradation on soil by* M. cladophyllus* UMAS MS8 were analysed and results were listed in Table 5. The results indicated a wide variation in RBBR dye degradation, from 1.99% to 91.01%, in the 12 trials. Runs 11 and 1 resulted in the highest RBBR dye degradation of 91.01% and 63%, respectively, at initial RBBR concentrations of 0.02 ppm. The Plackett-Burman design showed results that were generated by Design Expert 10.0.0 (Stat-Ease, Inc., Minneapolis, USA). The two values of each variable {maximum (+) and minimum (−)} were chosen such that the difference between the two values (+ and −) is large enough, as shown in Table 2.

The maximum RBBR dye degradation (Run 11) was obtained by having 8% lactose, 8% glucose, 0.02 ppm dye concentration, 13% (NH_4_)_2_SO_4_, 9% NH_4_NO_3_, 13% tryptone, 13% yeast, 9% NH_4_Cl, inoculum size of 1.0 cm, 80% moisture content, and incubation time of 15 days. Regression coefficients analysis and *t*-values of 5 factors (Table 6) were calculated and showed that *D*, *G*, *H*, *K*, and *L* had positive effects on RBBR dye degradation, whereas *A*, *B*, *D*, *E*, and *F* had negative effects. This positive effect indicated that these parameters will increase the RBBR dye degradation by increasing their concentration from low to high level. The variable with confidence level of above 95% is considered as significant parameters. Values of greater than 0.1000 indicated that the model is not significant. However, in this case, the model *F*-value of 22.44 implies that the model was significant since its value is largely greater than 0.1000. There is only a 0.08% chance that an *F*-value this large could occur due to noise. It was clear that variables *C*, *G*, *J*, and *L* were the significant factors, while *K*, with confidence levels below 95%, was considered as insignificant and thus was not selected.

The significant effects (factors and interactions) having *p* value less than 0.05 were selected as it is statistically different from zero at the 95% confidence level. On the other hand, the insignificant effects (factors and interactions) having *p* value higher than 0.05 were then excluded. Since the values of Prob > *F* less than 0.0500 indicated that the model terms are significant, thus in this case *C*, *G*, *J*, and *L* are the significant model terms. However, for this present study, only three variables (dye concentration, yeast concentration, and incubation time) were chosen since they were having the least number of *p* values. Moreover, for a better response surface method, it is advisable to have only three parameters.

Results obtained indicated that the ratio of C : N : P (35.8 : 40.8 : 1) was lower than the commonly used C : N : P ratio of 100 : 10 : 1 for soil bioremediation. Hence, addition of carbon and nitrogen source can accelerate decolourization of dye in soil. Yeast concentration was utilized as nitrogen source and it has been reported to influence the synthesis of ligninolytic enzymes, laccase, and peroxidase [21]. Yeast extract (organic compound) has been suggested to accelerate dye decolourization and important nitrogen source in decolourization of mix dye [22]. However, high concentration of nitrogen source can inhibit the production of fungal ligninolytic enzyme [23]. Since yeast extract appeared to be significant in this design,* M. cladophyllus* UMAS MS8 was expected to produce more laccase and peroxidase. Since there was a need for the addition of carbon, RBBR dye was thus utilized as the sole carbon source. Summary of statistics of the model including standard deviation, predicted residual sum of squares (PRESS), and coefficients of determination is presented in Table 7.

**Figure 2:**
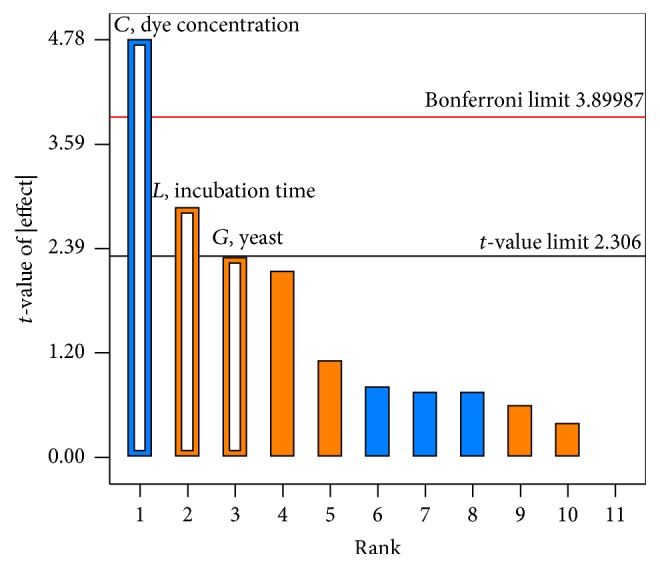
Pareto chart of RBBR dye removal in soil.* A*–*K* are the experimental variables as presented in Table 2.

The significance of the model examined by the determination coefficient (*R* ^2^) showed that more than 0.9492% of variance was attributable to the variables 29.03% of the total variance that cannot be explained by the model. The adjusted coefficient (*R* ^2^ adjusted) and predicted coefficient (*R* ^2^ predicted) were calculated to ensure the quality of fit of the polynomial model for RBBR bioremediation [24]. The *R* ^2^ Pred of 0.7969 was in agreement with the *R* ^2^ Adj of 0.90694. This indicated that the overall mean was a better predictor of the response than the current model. Adequate precision, which measures the signal to noise ratio, showed a value of 14.508 (>4 value is desirable) indicated an adequate signal. Thus, this model can be used to navigate the design space. The Pareto chart (Figure 2) offers a convenient way to view the results obtained by Plackett-Burman design and the order of significance of the variable affecting RBBR degradation. The vertical line in the chart defines the 95% confidence level.

The experimental values of RBBR degradation and theoretical values as predicted by Plackett-Burman design model equation showed a close agreement for all the medium components (Table 8). Errors represent the deviation value between predicted and actual values and were calculated based on (6) as follows:

(6) $$\begin{array}{c} \text{Error}=\frac{\text{Actual value}-\text{Predicted value}}{\text{Actual value}}\times 100\%. \end{array}$$

## 4. Conclusion

Based on Plackett-Burman design method, the work found that 4 (dye concentration, yeast extract concentration, inoculum size, and incubation time) out of the 11 factors investigated were the most effective factors on RBBR biodegradation in soil. However, only three variables (dye concentration, yeast extract concentration, and incubation time) were selected for further analysis since they have the least *p* values. Therefore, the Plackett-Burman design used in this work provided an efficient and rapid method for screening and selecting ingredients with a minimal number of experiments.

## Figures and Tables

**Table 1:** Remazol Brilliant Blue R specification (adapted from Mahmoud et al., 2014 [5]).

Synonym	Reactive Blue 19
Molecular formula	C_22_H_16_N_2_Na_2_O_11_S_3_
Molecular weight	626.54
CAS number	2580-78-1
Colour index number	61200
MDL number	MFCD00001215
Chemical structure	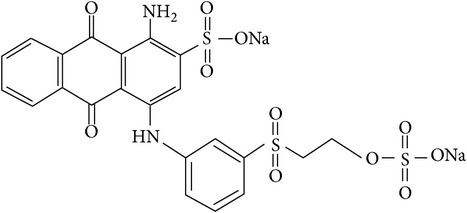

**Table 2:** Plackett-Burman range and the levels of the variables.

Independent variable (*X*_*i*_)	Range and levels	
	−*α*	+*α*
Lactose (%)	8	12
Glucose (%)	8	12
Dye concentration (ppm)	200	1000
Ammonium sulphate (%)	9	13
Ammonium nitrate (%)	9	13
Tryptone (%)	9	13
Yeast (%)	9	13
Ammonium chloride (%)	9	13
Inoculum size (cm)	0.2	1.0
Moisture content (%)	40	80
Incubation time (days)	3	15

**Table 3:** Experimental design layout for Plackett-Burman design.

Run	*A*	*B*	*C*	*D*	*E*	*F*	*G*	*H*	*I*	*J*	*K*
1	8	12	1000	9	13	13	13	9	3	40	1
2	12	12	1000	9	9	9	13	9	15	80	0.2
3	12	8	1000	13	9	13	13	13	3	40	0.2
4	8	12	1000	13	9	9	9	13	3	80	1
5	8	8	200	13	9	13	13	9	15	80	1
6	8	12	200	13	13	9	13	13	15	40	0.2
7	12	8	200	9	13	9	13	13	3	80	1
8	8	8	1000	9	13	13	9	13	15	80	0.2
9	8	8	200	9	9	9	9	9	3	40	0.2
10	12	8	1000	13	13	9	9	9	15	40	1
11	12	12	200	13	13	13	9	9	3	80	0.2
12	12	12	200	9	9	13	9	13	15	40	1

*A*: lactose (%), *B*: glucose (%), *C*: dye concentration (rpm), *D*: ammonium sulphate (%), *E*: ammonium nitrate (%), *F*: tryptone (%), *G*: yeast (%), *H*: ammonium chloride (%), *I*: incubation time (days), *J*: moisture content (%), and *K*: inoculum size (cm).

**Table 4:** Decolourisation of RBBR on agar plate by *M. cladophyllus *UMAS MS8 at 28°C.

Incubation time (days)	Concentration RBBR (%)		
	0.02	0.06	0.1
3	+	+	+
6	++	+	+
9	++	++	+
12	++	++	++
15	++	++	++

(+): partial or weak dye decolourisation; (++): complete dye decolourisation.

**Table 5:** Plackett-Burman experimental design for evaluating factors influencing dye degradation by *M. cladophyllus *UMAS MS8.

Run	*A*	*B*	*C*	*D*	*E*	*F*	*G*	*H*	*I*	*J*	*K*	Biodegradation
1	12	8	0.02	9	13	9	13	13	0.2	80	15	63.0
2	12	12	0.02	13	13	13	9	9	0.2	80	3	13.8
3	12	12	0.02	9	9	13	9	13	1.0	40	15	53.3
4	12	12	0.1	9	9	9	13	9	1.0	80	3	14.23
5	8	12	0.1	9	13	13	13	9	0.2	40	15	10.71
6	12	8	0.1	13	13	9	9	9	1.0	40	15	12.54
7	8	12	0.02	13	13	9	13	13	1.0	40	3	51.31
8	8	8	0.1	9	13	13	9	13	1.0	80	3	6.03
9	8	8	0.02	9	9	9	9	9	0.2	40	3	20.28
10	8	12	0.1	13	9	9	9	13	0.2	80	15	15.53
11	8	8	0.02	13	9	13	13	9	1.0	80	15	91.01
12	12	8	0.1	13	9	13	13	13	0.2	40	3	1.99

*A*: lactose (%), *B*: glucose (%), *C*: dye concentration (ppm), *D*: ammonium sulphate (%), *E*: ammonium nitrate (%), *F*: tryptone (%), *G*: yeast (%), *H*: ammonium chloride (%), *I*: inoculum size (cm), *J*: moisture content (%), and *K*: incubation time (days).

**Table 6:** Analysis of variance for the regression model and respective model terms.

Source	Sum of squares	DF^*∗*^	Mean square	*F*-value	Prob > *F*	Remarks
Model	8216.67	5	1643.33	22.44	0.0008	Significant
*C*, dye concentration	4472.58	1	4472.58	61.08	0.0002	
*G*, yeast	1022.50	1	1022.50	13.96	0.0097	
*J*, inoculum size	885.97	1	885.97	12.10	0.0132	
*K*, moisture content	238.25	1	238.25	3.25	0.1213	
*L*, incubation time	1597.37	1	1597.37	21.81	0.0034	
Residual	439.34	6	73.22			
Cor total	8656.02	11				

^*∗*^DF = degree of freedom.

**Table 7:** Analysis of variance for the regression model and respective model terms.

Parameters	Value
Standard deviation	8.56
Mean	29.48
Coefficient of variation (CV) (%)	29.03
*R* ^2^	0.9492440752809
Adjusted *R* ^2^	0.90694747134832
Predicted *R* ^2^	0.7969763011236
Predicted residual sum of squares (PRESS)	1757.377
Adequate precission	14.508

**Table 8:** Actual and predicted values for RBBR dye degradation.

Run order	Actual value	Predicted value	Error (%)
1	63.00	65.42	−3.841
2	13.80	23.88	−73.04
3	53.30	55.23	−3.62
4	14.23	20.91	−41.88
5	10.71	17.89	−67.0
6	12.54	16.61	−32.46
7	51.31	50.61	1.36
8	6.03	2.45	59.37
9	20.28	14.97	26.18
10	15.53	8.34	46.3
11	91.01	82.60	9.24
12	1.99	−5.18	−160.3
